# Marine Resources Gels as Main Ingredient for Wound Healing Biomaterials: Obtaining and Characterization

**DOI:** 10.3390/gels10110729

**Published:** 2024-11-11

**Authors:** Alina Elena Coman, Maria Minodora Marin, Ana Maria Roșca, Madalina Georgiana Albu Kaya, Rodica Roxana Constantinescu, Irina Titorencu

**Affiliations:** 1 Department of Collagen, Division of Leather and Footwear Research Institute, National Research and Development Institute for Textiles and Leather, 93 Ion Minulescu Str., 031215 Bucharest, Romania; coman.alina27@yahoo.com (A.E.C.); albu_mada@yahoo.com (M.G.A.K.); rodica.roxana@yahoo.com (R.R.C.); 2 Advanced Polymer Materials Group, University POLITEHNICA of Bucharest, 1-7 Polizu Street, 011061 Bucharest, Romania; 3 Institute of Cellular Biology and Pathology ‘’Nicolae Simionescu’’, 8 B. P. Hasdeu Street, District 5, 050568 Bucharest, Romania; irina.titorencu@gmail.com

**Keywords:** fish wastes, marine collagen, biomaterials, wound healing

## Abstract

The skin, known as the largest organ of the body, is essential for maintaining physiological balance and acts as a barrier against the external environment. When skin becomes damaged and wounds appear on the skin’s surface, a complex healing process, involving multiple types of cells and microenvironments, take place. Selecting a suitable dressing for a wound is crucial for accelerating healing, reducing treatment costs, and improving the patient’s overall health. Starting from natural resources such as perch skin (*P. fluviatilis*), this article aims to develop biocompatible materials for regenerative medicine from collagen in the form of gels/gelatines. The extracted gels were physical/chemical and structurally analyzed. In order to obtain collagen scaffolds for wound healing, the extracted collagen gels from perch skin were further freeze-dried. The ability of these scaffolds is essential for controlling moisture levels during wound healing; therefore, it was necessary to investigate the samples’ ability to absorb water. The assessed collagen-based scaffolds were microbiologically tested, and their biocompatibility was investigated by incubating human adult dermal fibroblasts. The outcomes reveal an innovative path for the production of biomaterials used in wound healing, starting from collagen derived from marine sources.

## 1. Introduction

In recent decades, the researchers starting to focus on new potential collagen sources due to the rising demand of high purity collagen and the limitation of porcine and bovine collagen extraction. Usually, collagen is extracted from porcine or bovine, but there are some limitations, such as the possibility of disease transmission and socio-cultural and religious concerns [1,2,3,4].

Despite mammalian collagen extraction, more efficient and sustainable sources should be exploited in order to meet the market demand [5,6]. Globally, around 20 million tons of aquaculture wastes are generated, and nearly 25% are fish wastes, an important source of high purity collagen. Fish wastes are generated in high quantities every year due to the human consumption. This includes skin, scales, viscera, bladders, ligaments and bones, representing around 75% of a fish’s weight. Usually, fish wastes are burned or buried, leading to environmental, economic and health problems. A minor percentage is employed as raw materials in feed for animals [7,8,9,10].

Therefore, fish by-product valorization, as potential collagen sources, solve ecological and economic issues, leading to rentability in terms of profitability. Moreover, collagen derived from marine sources is religiously accepted by various groups [2,3,4].

In terms of properties, the denaturation temperature of marine collagen is lower, being in the range of 25–30 °C for most fish species (depending on the composition), in comparison with mammalian collagen, which has a denaturation temperature of 39–40 °C [4,11]. However, collagen derived from fish and invertebrate species is more bioavailable and is absorbed up to 1.5 times better than collagen from cows or pigs [12]. For example, faster blood circulation is ensured by small particle size and low molecular weight for marine collagen [13]. Also, fish collagen posed higher flexibility and a similar viscosity compared with mammalian collagen [4]. Furthermore, the amino acid content and biocompatibility of marine collagen are comparable to those of bovine and pig collagen [14].

Fish skin is a natural raw material that contains minerals, vitamins, proteins and especially collagen. Its features include improved physical/chemical properties, superior biocompatibility, easy processing, high porosity, and the capability to combine with synthetic and natural materials, which increased researchers’ attention regarding fish skin. Type I collagen is known to be the most abundant protein in fish skin and in the human body.

In addition, fish skin contains omega-3 polyunsaturated fatty acids, which improves the wound healing process [3,4,15]. Thereby, wound dressing from fish wastes can be successfully used.

The largest organ of the body, the skin, plays a crucial role in preserving physiological equilibrium and providing defense against the outside world. Numerous cell types and microenvironments are involved in a complex healing process when skin is damaged and wounds appear on the skin surface. Selecting the right wound dressing is essential to promote healing, lower treatment expenses, and enhance the patient’s general health. However, the variety of wound dressings on the market can be confusing. In order to avoid negative outcomes, wound care must be treated properly. Wound dressings are primarily used to create a transient physical barrier of protection, absorb wound drainage and supply the moisture required to maximize re-epithelialization. The wound’s anatomical and pathophysiological features influence the dressing selection. Additional advantages of contemporary wound dressings include pain alleviation and antibacterial qualities [16,17,18].

The objective of this paper is represented by the development of biocompatible materials for regenerative medicine starting from collagen gels/gelatines, using natural resources such as fish skin. This research focuses on collagen extracted from perch skin (*P. fluviatilis*) in the form of a gel. To the best of our knowledge, there is no existing literature or research that addresses collagen extracted from perch skin for regenerative medicine purposes, leading to the novelty of these research studies.

The gels extracted were analyzed by proximate analysis (dry substance, protein and ash content, total nitrogen, collagen yield and pH), FT-IR spectroscopy and circular dichroism (CD).

The extracted collagen gels from perch skin were further freeze-dried in order to obtain collagen scaffolds for wound healing. Since these scaffolds are essential for controlling moisture levels during wound healing [19], the water uptake capacity of the samples obtained was investigated. The resulting collagen biomaterials were also microbiologically characterized. Fibroblasts are essential for the proper healing of wounds, having a crucial role in processes such as contracting the wound; producing new collagen and extracellular matrix structures in order to support other cells involved in successful wound healing; and decomposing the fibrin clot [20]. For this reason, the biocompatibility of the tested collagen-based scaffolds was evaluated by incubating human adult dermal fibroblasts.

The results reveal a new opportunity for the development of biomaterials employed in wound healing, starting from collagen of marine origin.

## 2. Results and Discussion

### 2.1. Characterization of Collagen Gels Extracted from Perch Skin

Collagen gels extracted from perch skin through various treatments and at different temperatures were physically/chemically and structurally analyzed.

#### 2.1.1. Physical/Chemical Analysis

For a physical/chemical analysis of the extracted gels, a proximate analysis was performed, and the results are presented in Table 1.

Between the samples, there are some differences regarding the storage temperature during the extraction. For the samples treated with tartaric and citric acid, higher values are obtained for dry substances (represented by collagen quantity obtained after extraction, reported at 100 g, with the difference being water [21]), and for protein content (a high protein content leads to high amounts of amino acid being transformed into collagen and increased sample purity) for samples stored at room temperature, noticing the temperature influence.

The dry substance and protein content are high for all the samples, leading to adequate values for gel samples with high water content.

The pH obtained is acidic, 2.5 for all the samples, which is very good for collagen deterioration prevention and stability assurance [22].

For samples with ascorbic acid used for extraction, the results obtained for samples kept in the refrigerator are slightly higher compared with the ones kept at room temperature.

Collagen extraction is predominantly based on the treatment of fish skin in order to reduce non-collagenous components such as minerals, lipids and other impurities. The removal of non-collagenous materials occurs during the overall extraction process, starting with the pretreatment of the collagen source, and its extraction and purification process [23]. The samples’ purity increased for all the samples and is demonstrated by the ash content [24], which could not be detected in any of the samples.

GEL_COLL_A_f_ presents the highest protein and dry substance content from samples kept in the fridge. These results will also be further confirmed by circular dichroism.

**Figure 1:**
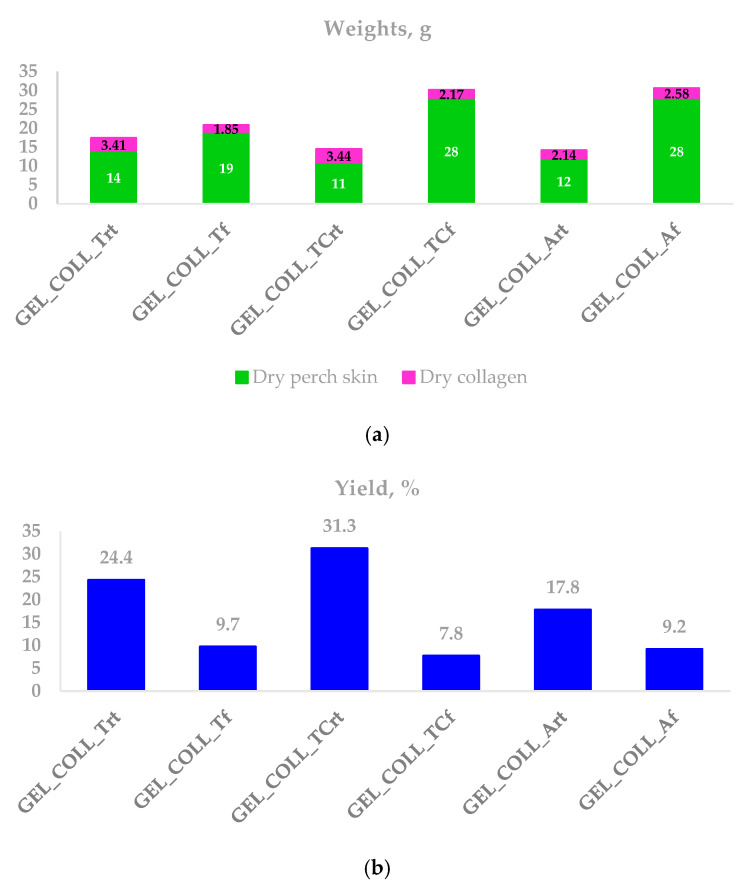
Yields of collagen extracted from perch skin. (**a**) Dry weights (in grams) of collagen extracted from the indicated weight of skin. (**b**) Collagen yields of the samples are calculated as (Dry collagen weight/Dry skin weight) × 100.

The dry substance content was used to determine the yields of collagen extraction. The dry substance is represented by the dry collagen content from the sample. Figure 1 shows the yields obtained for collagen gel extracted from perch skin. The yield is higher for all the samples kept at room temperature, compared with samples stored in the fridge.

The yields reached for collagen gel through acid extraction from *P. fluviatilis* skin are very good compared with the yields obtained in other studies. The yield of *Dasyatis zugei* skin collagen was reported to be higher when acetic acid was used for extraction (approx. 11.49%) compared with using hydrochloric acid (approx. 3.50%) [25]. Different yields were obtained for *Pangasius* sp. skin collagen extracted with 0.5 M and 0.7 M acetic acid (approx. 10.94% and 5.47%, respectively) [26]. On the contrary, *Decapterus macrosoma* collagen extracted with 0.5 M and 0.7 M acetic acid were approx. 1.01% and 1.31, respectively, which means a higher yield percentage was obtained with 0.7 M acetic acid [27].

#### 2.1.2. Structural Analysis

- *Circular Dichroism Spectroscopy*

**Figure 2:**
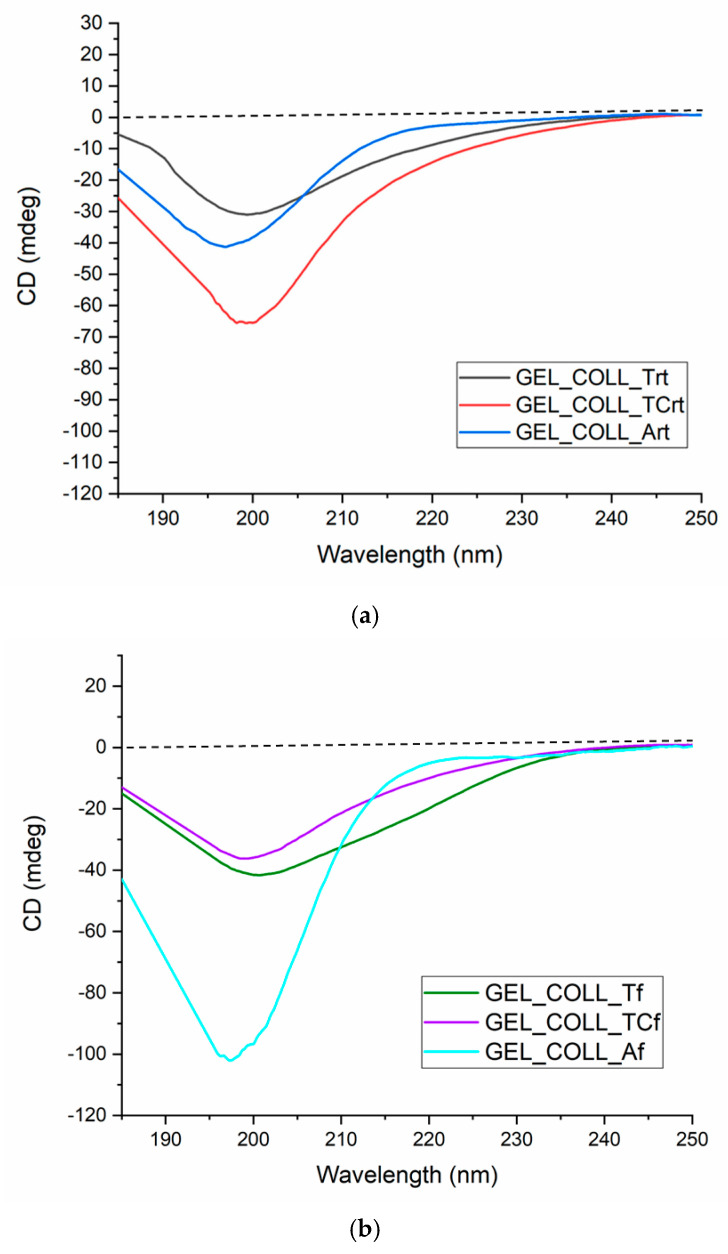
Circular dichroism spectra for perch gels; (**a**) samples kept at room temperature, (**b**) samples kept in the fridge.

Circular dichroism spectroscopy (CD) is presented in Figure 2 for all the collagen samples from perch skin. CD spectra are similar for all the samples, with a pronounced negative band around 196 to 200 nm, suggesting a random coil structure and no positive maximum around wavelength of 220 nm, specific to the triple-helix structure of collagen.

According to the literature, after complete collagen denaturation, the positive band specific to the triple-helix structure (between 220 and 230 nm) disappears and only the negative band remains around 200 nm, specific to gelatine [28,29,30]. Therefore, for all collagen gels extracted, were assigned a structure specific to denatured collagen, namely the gelatine structure.

Studies performed by Gopinath et al. [31] present a connection between the protein denaturation process, the maximum positive peak and minimum negative peak.

Analyzing the CD spectra profiles for all the samples, from Figure 2, and assuming the presumption that a higher ratio between the peaks, as mentioned before, we can conclude that GELL_COLL_T_rt_ is the most denatured extract from perch skin, where tartaric acid was used for extraction, and the sample was kept at room temperature (the experiments were performed during the winter, at maximum 15 °C). The less denatured sample is GELL_COLL_A_f_, where ascorbic acid was used for extraction and samples were kept in the refrigerator (4 °C); therefore, the negative bands with higher values indicate a lower denaturation. Once again, the temperature influence upon conformational structure in this case is demonstrated. The prevalence of random coil structures and no bands characteristic of the triple-helix structure, which are specific to collagen, are in concordance with the FTIR spectra, presented further, demonstrating that the perch skin collagen extracts have a gelatinous structure.

Moreover, Echave et al. [32] demonstrate that the use of denatured collagen and gelatine increases cell growth, promoting the biological response of medical devices used in tissue engineering.

- *Fourier transform infrared spectroscopy (FTIR)*

**Figure 3:**
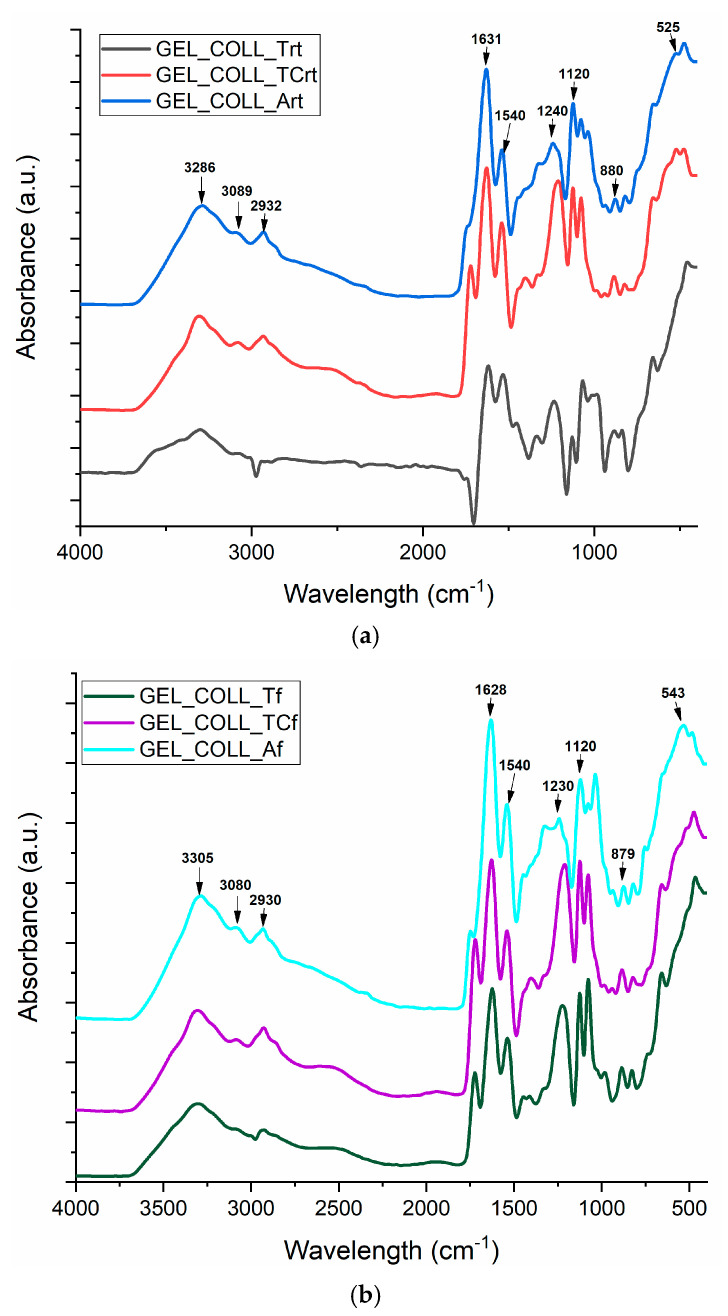
FTIR spectra of perch gels; (**a**) samples kept at room temperature, (**b**) samples kept at fridge.

Gels extracted from perch skin present spectral bands characteristic of the functional groups in the collagen structure, such as amide A, B, I, II and III. In Figure 3, there are no significant differences between samples. The signal of the peaks between 3305 and 3280 cm^−1^ is represented by the amide A structure and is specific to the stretching vibrations of N-H groups coupled with hydrogen bonds from the carbonyl group in the peptide chain. Amide B presents signals in the range of 3090–2930 cm^−1^, with the stretching vibration of the methylene group being symmetrical and asymmetrical. The peak characteristic of amide I at approximately 1631 cm^−1^ is similar to other fish extracts [33,34,35].

Amide I, specific to the secondary structure of proteins, with a peak characteristic of carbonyl group stretching vibration in the peptide structure, presents an intense absorption at 1660 cm^−1^, attributed to the collagen triple-helix structure; otherwise, the intensity decreases and the band intensification around 1630 cm^−1^ is associated with collagen denaturation, resulting gelatine [36].

The bands characteristic of amides II and III represent the bending vibrations of the N-H group (at 1540 cm^−1^) coupled with the stretching vibrations of the C-N and C-H groups (1240–1210 cm^−1^). In the case of collagen, the specific intensity of the amide II peak is more intense, around 1550 cm^−1^, so the FTIR spectra clearly indicate the gelatine structure. The peaks at 1120 cm^−1^ represent the asymmetric bonds of CO-O-C glycogen and nucleic acids. The peaks appearing at wave numbers between 880 and 525 cm^−1^ represent the protein skeleton [37,38].

### 2.2. Characterization of Collagen Scaffolds

Following the characterization of perch gels, the best properties were obtained for samples stored in a refrigerator, despite having the lowest yield, based on physical/chemical properties and circular dichroism, which showed a lower denaturation of perch gel samples; therefore, the samples kept in a refrigerator were chosen for further analysis. The perch gels selected were poured into a glass Petri dish and freeze-dried for 48 h [35] in order to obtain collagen scaffolds (spongious forms), which were further tested to determine the water absorption, conduct a microbiological analysis and determine the biocompatibility of the synthesized collagen scaffolds.

#### 2.2.1. Water Absorption of Collagen Scaffolds

**Figure 4:**
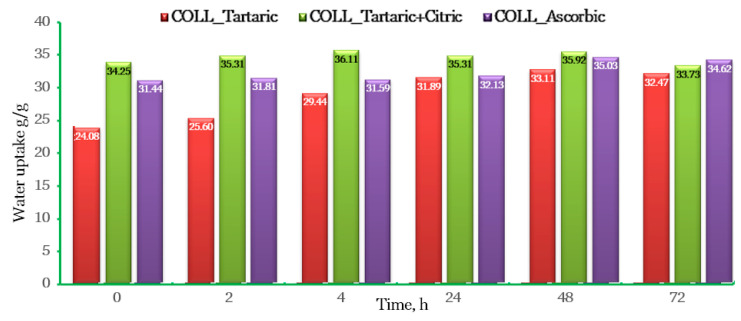
Water uptake of biocomposites.

The water uptake was estimated for three days at different intervals of time and showed that all the samples were stable, as shown in Figure 4.

The sample COLL_TC absorbed the highest amount of water, about 36 g/g water in the first 48 h. COLL_A seems to be denser, therefore absorbing a smaller amount of water, around 35 g/g water in the first 48 h. COLL_T absorbed around 33 g/g water.

All the samples present stability in the first 48 h, and then the samples start to dissolve.

Prior to selecting a wound dressing, it is critical to consider a number of criteria, including the wound dressing’s cost, convenience of administration, duration of therapy and capacity to absorb exudate in an effective manner, along with other factors. Hence, water absorption is a key factor in the development of wound dressing.

#### 2.2.2. Collagen Scaffolds Microbiological Analysis

Microbiological testing is imperative for patient health protection. Medical device manufacturers must ensure that their products meet the highest quality control standards. Microbiological testing is essential to ensure that a device is safe and effective by examining the presence of harmful pathogens that could contaminate it [39].

**Figure 5:**
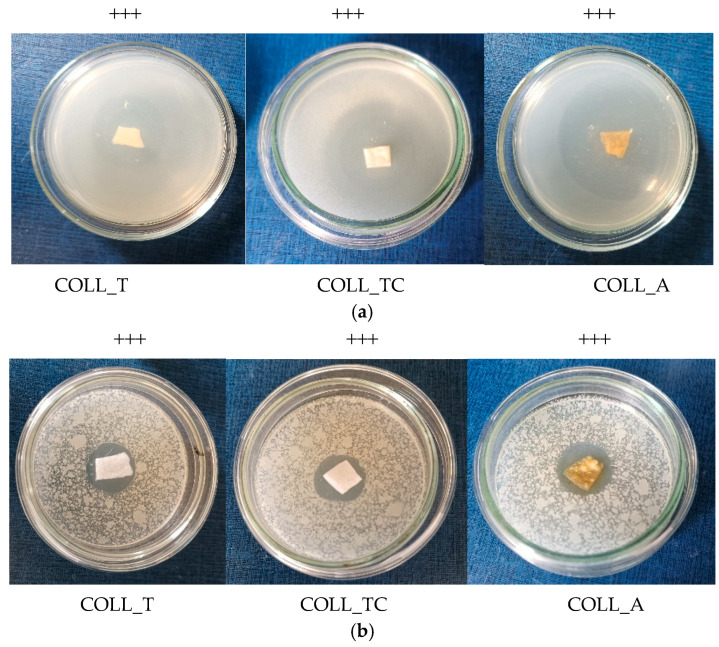
Antimicrobial activity of perch sponges against (**a**) *E. coli* and (**b**) *S. aureus*.

From Figure 5, it can be observed that all the samples presented antibacterial activity against *Escherichia coli* (*E. coli*) and *Staphylococcus aureus* (*S. aureus*), with intense activity on *E. coli*, which proves the efficiency of these collagen scaffolds for medical applications use.

The difference between the degree of inhibition for the two types of bacteria tested may be due to the structure of the cell wall. *E. coli* is a Gram-negative bacteria unlike *S. aureus*, which is a Gram-positive bacteria with a more resistant cell wall due to the basic unit—peptidoglycan.

Depending on the diameter of the inhibition zones formed, the results are interpreted as follows:

- 0–10 mm, inactive—marked "−";
- 10–14 mm, weak activity—noted "+";
- 15–19 mm, moderate activity—marked "++";
- ≥20 mm, definite activity—marked “+++”.

The scope of microbial contamination determination, in accordance with the provisions of the European Pharmacopoeia Ed. 10/20, is to identify microbial contamination of the samples (Table 2). Microbial contamination control aims to determine the total number of aerobic microorganisms or the absence of pathogenic microorganisms:

- Total number of aerobic microorganisms (TAMC);
- Total number of yeasts and filamentous fungi (TYMC).

The bacteria tested on collagen scaffold were Escherichia coli (*E. coli*), Staphylococcus aureus (*S. aureus*), and Pseudomonas aeruginosa (*P. aeruginosa*).

From the obtained results, during the microbiological characterization of the tested collagen scaffolds, it can be observed that the obtained the values do not exceed the limits of admissibility provided by the European Pharmacopoeia, the currently used edition. The samples do not allow the development of aerobic germs for any of the tested bacteria [40,41,42,43].

#### 2.2.3. Collagen Scaffolds Biocompatibility 

The biocompatibility of the tested collagen-based scaffolds was assessed by incubating human adult dermal fibroblasts with extracts obtained as described in the Materials and Methods. Since the dermal fibroblasts are one of the major players in the wound healing process, we first looked at their viability in the presence of scaffold-based extracts. Moreover, given the fact that fibroblast motility is an important feature of these cells, which should be able to migrate from the areas surrounding the lesion into the injury site and colonize the scaffolds, we subsequently verified whether this ability of human dermal fibroblasts was impacted by the scaffolds.

**Figure 6:**
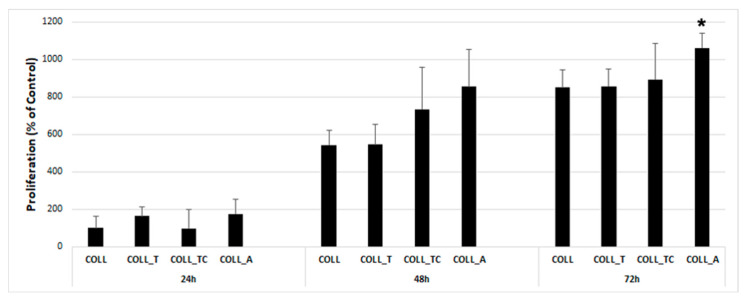
XTT assay showing the viability of human adult dermal fibroblasts after 24, 48 and 72 h of incubation in the presence of collagen scaffold extracts (* *p* < 0.05 versus COLL at 72 h).

As shown in Figure 6, no cytotoxic effect was observed for the tested scaffold extracts. Furthermore, the data indicated that, at 48 h, the COLL_T and COLL_TC scaffold sustained a slightly increased viability compared to the COLL scaffold extract; however, no statistical significance was obtained. At 72 h, the viability was similar for all extracts, except for COLL_A, which was 20% higher than the COLL extract. These data suggest that none of the tested scaffolds had any cytotoxic effect; moreover, COLL_A could have a positive impact on cell viability to some extent, possibly by promoting cell proliferation.

**Figure 7:**
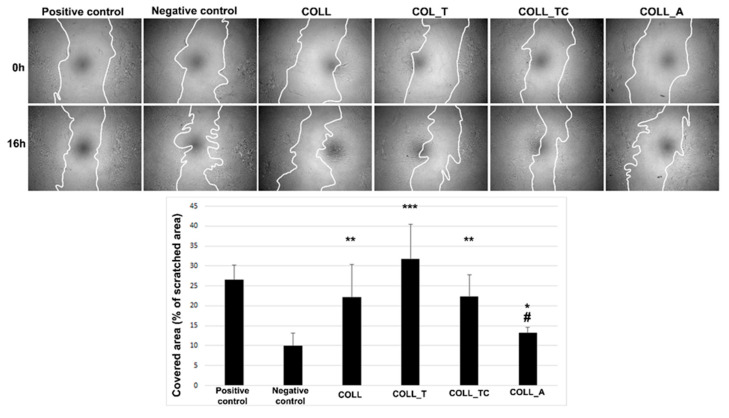
In vitro scratch test showing the impact of collagen scaffold extracts on the fibroblast motility: upper panel—phase-contrast microscopy showing the scratched area at 0 h and 16 h later (magnification 5×), lower panel—the quantification of covered area as percentage of the initial scratched area where positive and negative controls are serum and serum-free medium (* *p* < 0.05; ** *p* < 0.005, *** *p* < 0.001 versus negative control, # *p* < 0.05 versus COLL).

As mentioned above, the migration of cells, including dermal fibroblasts, at the lesion site is an important step in the wound healing progression. Moreover, in the case of using these scaffolds for wound healing, the migration of fibroblasts in order to colonize biomaterials should not be inhibited. Therefore, we evaluated the impact of the tested scaffold extracts on this key feature. Thus, Figure 7 indicates that all extracts promoted fibroblast migration in comparison to the negative control, which was a serum-free medium (controls used in order to verify that the technique was correctly performed). No statistical significance was obtained when compared to the COLL extract, with the exception of the COLL_A extract, which induced a cell migration about 40% lower than the COLL control.

**Figure 8:**
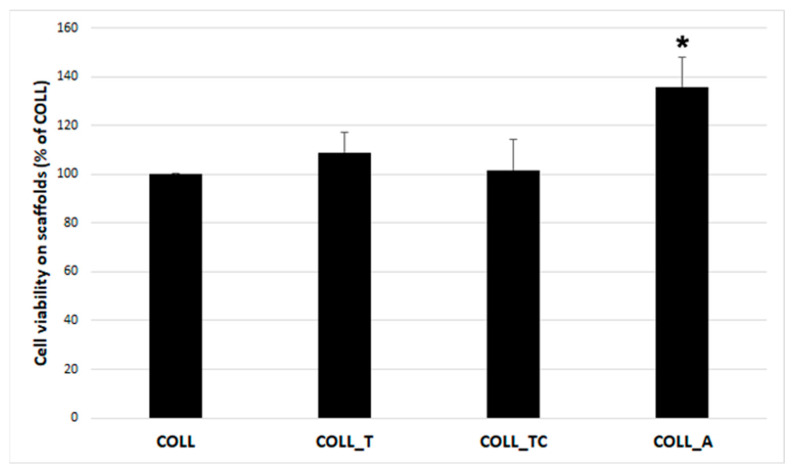
XTT assay showing the viability of human adult dermal fibroblasts 5 days after seeding onto collagen scaffolds (* *p* < 0.05).

Next, the capacity of the tested scaffolds was assessed to support colonization with dermal fibroblasts. For this, cells were grown on these samples as described above and viability was evaluated. As seen in Figure 8, the viability for cells grown on the COLL_T and COLL_TC scaffolds was similar to those grown on the COLL scaffold. However, in the case of the COLL_A scaffold, the viability was 35% higher than the COLL control. This corroborates well with the previous data regarding the lack of toxicity of all extracts and the increased viability in the presence of COLL_A extract 72 h after seeding in 2D culture.

**Figure 9:**
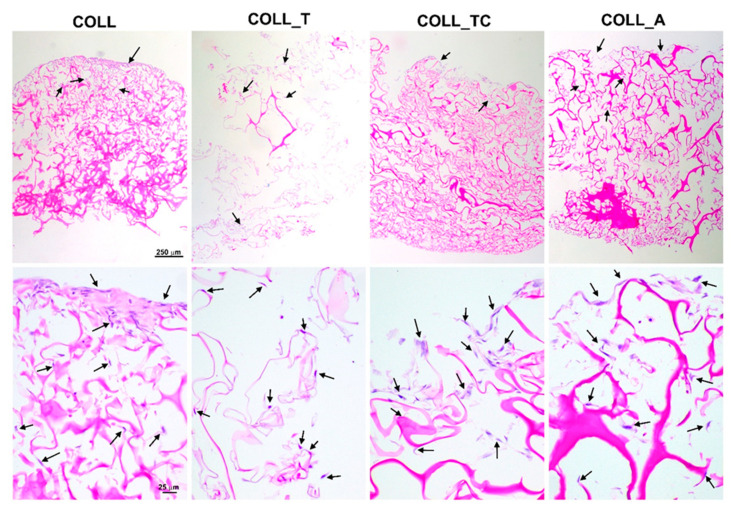
Hematoxylin and Eosin staining illustrating the colonization of collagen scaffolds with human dermal fibroblasts. The presence of cells attached to the collagen fibers is indicated by arrows.

The Hematoxylin and Eosin histological staining showed that all scaffolds sustained three-dimensional growth of dermal fibroblasts, both at the surface and inside the scaffolds, within the structural pores (Figure 9). In the case of COLL_T, the structure is lax, most likely due to partial degradation in the presence of cells. All the other scaffolds retained integrity in the given timeline.

**Figure 10:**
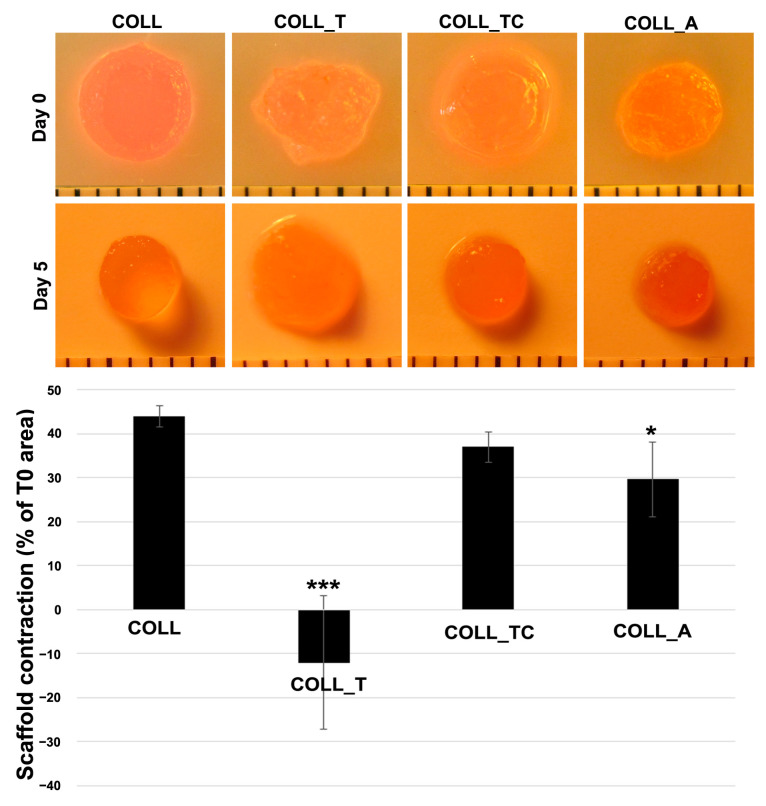
Quantification of collagen scaffolds contraction: upper panel—images of the scaffolds before seeding of fibroblasts and after 5 days in culture; lower panel—quantification of the contracted area (* *p* < 0.05, *** *p* < 0.001). Each image was individually calibrated.

Next, we checked whether the various collagen scaffolds support the ability of dermal fibroblasts to perform contraction. In Figure 10, it can be noticed that the area of the COLL-T scaffold increased after 5 days in the presence of cells. Keeping in mind that the histological analysis showed a certain degradation of collagen scaffold in the presence of fibroblasts, we assume that this increase in size and lack of contraction is due to the loss of structural integrity following the secretory activity of the cultured cells. In contrast, the other two scaffolds, COLL_TC and COLL_A, were less contracted by fibroblasts in comparison with COLL, although the difference was statistically significant only for the COLL_A scaffold. Thus, the latter showed a contraction about 25% lower than COLL.

Our data showed that all tested scaffolds were biocompatible, and COLL_A was capable of promoting a higher viability of fibroblasts in comparison with the COLL control and the other tested scaffolds, both in the case of extracts obtained by incubation in a complete culture medium and by direct contact with human dermal fibroblasts, suggesting a higher capacity to sustain colonization. Although the COLL_A extract promoted dermal fibroblast migration to a smaller extent than the COLL extract, as shown by the scratch test assay, this might not be the case in vivo, keeping in mind that the extracts were obtained in the absence of cells. Interestingly, the histological analysis showed a certain degradation of the COLL_T scaffold after 5 days in the presence of human dermal fibroblasts, suggesting that this particular biomaterial could not be appropriate for in vivo use, since the degradation of the scaffold should be synchronized with the regeneration process in order to ensure support for the migration and proliferation of cells from the surrounding tissue. All the other tested scaffolds were stable for up to 5 days in the culture and in the presence of cells. Since, throughout the wound healing process, the proliferative phase begins during the first week after injury [44,45], the scaffold should be stable in a biological environment at least for this period of time. As the fibroblasts proliferate and begin to secrete extracellular matrix, the scaffold should be able to be slowly degraded under the activity of enzymes in order to make place for the newly formed tissue. Our results indicated that, with the exception of COLL_T, all the tested scaffolds retained their structure for at least 5 days in the presence of dermal fibroblasts, suggesting they are suitable for in vivo use [44,45].

Furthermore, we examined the capacity of the scaffolds to support dermal fibroblast contraction. It is known that although this process is normal during wound healing, having the role of pulling together the edges of the wound to minimize its size in the case of large wounds could lead to complications, such as scarring and fibrosis or even the loss of a tissue function [46]. Thus, efforts are being made to create biomaterials that inhibit or do not support a high degree of contraction to circumvent these disadvantages [47]. Data showed that, with the exception of the COLL_T scaffold, which appeared to increase in size after 5 days in culture in the presence of dermal fibroblasts most likely due to degradation, COLL_TC and COLL_A exhibited a lower contraction in comparison to the COLL scaffold, which was statistically significant for COLL_A. Thus, corroborating these data, we conclude that the COLL_A scaffold could be a valuable tool for wound healing.

## 3. Conclusions

This paper highlights the development of biocompatible materials for regenerative medicine starting from natural resources, such as fish skin. This research focuses on collagen extracted from perch skin (*P. fluviatilis*) in the form of a gel in order to obtain collagen scaffolds for wound healing.

The resulting collagen gel samples were analyzed by proximate analysis. The sample with ascorbic acid used for extraction and stored in the refrigerator (GEL_COLL_Af) presents the highest protein and dry substance content. The results obtained were also confirmed by circular dichroism (CD).

It can be concluded from CD spectra profiles that the most denatured sample from perch skin was GELL_COLL_Trt, with tartaric acid used for extraction and stored at room temperature (the studies were conducted during the winter, at a maximum room temperature of 15 °C). When ascorbic acid was used for extraction and the sample was kept in the refrigerator (4 °C), GELL_COLL_Af was less denatured; as a result, the negative bands with higher values indicate a lesser denaturation. At this point, it is demonstrated how temperature affects conformational structure.

The FT-IR spectra present the random coil structure preponderance and the absence of any band distinctive of the triple-helix structure specific to collagen. This indicates that the collagen extracts from perch skin have a gelatinous structure, which is in concordance with CD spectra.

The best properties of the analyzed perch gels were observed in samples stored in the refrigerator, especially for GELL_COLL_Af, despite the lowest yield; therefore, the samples kept in the refrigerator were further freeze-dried in order to obtain collagen scaffolds (spongious forms), which were further tested to determine the water absorption, conduct a microbiological analysis and analyze the biocompatibility of the synthesized collagen scaffolds. All three samples (COLL_T, COLL-TC, COLL_A) present stability in the first 48 h, and then they start to dissolve.

From the microbiological analysis, it was observed that all the samples presented antibacterial activity against *Escherichia coli* (*E. coli*) and *Staphylococcus aureus* (*S. aureus*), with intense activity on *E. coli*, which proves the efficiency of collagen scaffolds for wound dressing. The samples do not allow the development of aerobic germs for any of the tested bacteria.

The biological data demonstrated the biocompatibility of all tested scaffolds, and the COLL_A sample was able to support fibroblast viability that was higher than that of the control (COLL sample) and the other tested scaffolds, both for extracts obtained by direct contact with human dermal fibroblasts and for extracts obtained by incubation in complete culture medium, indicating a higher capacity capable to sustain colonization. Despite the fact that the scratch test assay demonstrated that the COLL_A extract facilitated dermal fibroblast migration more than the COLL sample, this may not be the case in vivo, taking into account that the extracts were produced without cells. Moreover, the capacity of the scaffolds to support dermal fibroblast contraction was examined. The contraction process is normal during wound healing. The data resulting from this analysis showed that, with the exception of the COLL_T scaffold, which appeared to increase in size after 5 days in culture in the presence of dermal fibroblasts likely due to degradation, COLL_TC and COLL_A exhibited a lower contraction in comparison with the COLL scaffold, which was statistically significant for COLL_A. Hence, it can be concluded that the COLL_A scaffold could be a valuable tool for wound healing, revealing a new opportunity for biomaterials used in regenerative medicine, starting from collagen of marine origin.

## 4. Materials and Methods

### 4.1. Process of Acidic Collagen Extraction

**Figure 11:**
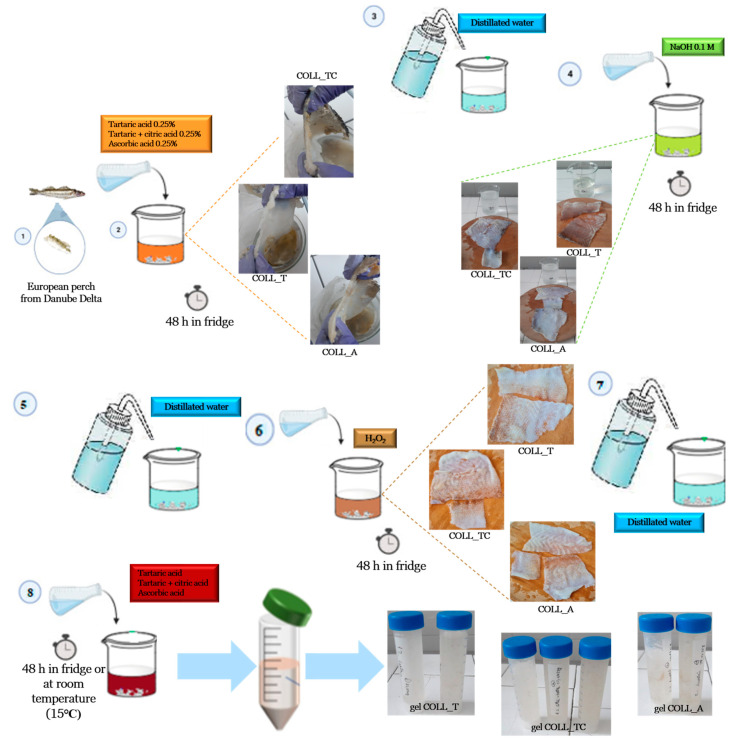
Schematic representation of acidic extraction of collagen gel from perch skin.

The fresh European perch skin was bought from a local fishing company, Tulcea, Danube Delta, Romania. In our country, perch consumption is very high, leading to large quantities of fish wastes. In this context, we tried to find a viable solution in order to valorize the by-products, which, in this case, was perch skin. The fresh skin was frozen until processing. After defrosting, the skin was washed thoroughly with bio detergent and cold distilled water in the first step. After washing, several treatments followed in order to obtain collagen gels. Figure 11 schematically shows all the treatment stages subjected to the skin, which are detailed further.

After washing, the perch skin samples were placed in three different acidic solutions with 0.25% concentration of acids and kept in the refrigerator for two days. The acids used were L (+) tartaric acid (Chimreactiv, Bucharest, Romania), L (+) tartaric and citric acid (Chimreactiv, Bucharest, Romania) in equal proportions and L (+) ascorbic acid (Scharlau, Sentmenat, Spain). After two days, it can be seen the skin is slightly swollen.

In the third stage, the samples were washed with distilled water until a neutral pH to avoid precipitation of acids remained when adding a solution of 0.1 M sodium hydroxide (Chemical Company, Iasi, Romania) in the fourth stage. In the alkaline treatment, the samples were kept in the refrigerator for 48 h. Sodium hydroxide helps to remove non-collagenous proteins, as can be seen in the pictures.

During stage five, the samples were washed with distilled water, followed by stage six, the process of pigment removal from the skin. For this, the samples were placed in a solution of 2% hydrogen peroxide (hydrogen peroxide solution 30%, Silal Trading, Bucharest, Romania) and kept 48 h in the refrigerator.

After washing the samples with distilled water, the last step for collagen gel extraction from perch skin is the addition of the acids used initially in the acidic treatment. In order to see the temperature influence on sample’s properties, the treated skin was split in two: one part stayed in the refrigerator for 48 h, and the other stayed at room temperature (15 °C). The final results were collagen gels illustrated in Figure 11.

**Figure 12:**
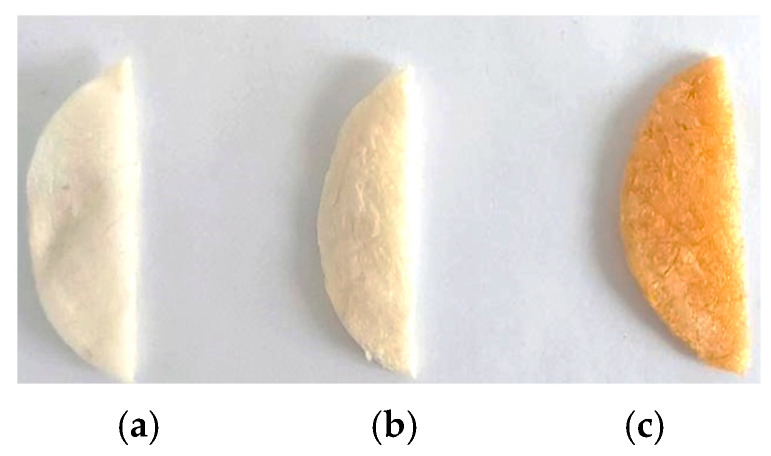
Collagen scaffolds, gels after freeze drying, (**a**) COLL_T, (**b**) COLL_TC and (**c**) COLL_A.

The pH of perch gels extracted was adjusted from 2.5 to 7.2–7.4 (for promoting cell growth [48]) with NaOH 0.1 M and afterward, the gels were poured into a glass Petri dish and freeze-dried for 48 h [49] to obtain collagen scaffolds (spongious forms, Figure 12).

### 4.2. Physical/Chemical Analysis and Extracted Collagen Yields

Dry substance, ash and protein contents of extracted gels were determined according to standard methods SR EN ISO 4684:2006, SR EN ISO 4047:2002 and SR ISO 5397:1996. Total nitrogen was determined by the Kjeldahl method. The pH was determined potentiometrically according to SR EN ISO 4045:2008.

The yield of extracted collagen was calculated on basis of the weight of fresh skin:

% Yield = (Dry substance content weight/Dry skin weight) × 100
(1)

where *Dry substance content weight* represents dry collagen.

### 4.3. Water Uptake of Collagen Scaffolds

The water uptake capacity of collagen sponges was determined by weight method [16], which involves weighing the sponges before and after immersion in water. A piece of sponge of about 1 cm^3^ was weighed at reference time and then placed in 3 mL water at room temperature (about 23 °C). At defined periods of time, the sponges were weighed and the capacity to retain water was calculated using the following equation (Equation (2)):

Water uptake (%) = [(Wt − Wd)/Wd] × 100
(2)

where *Wt* represents the weight of water retained by sponges at time t and *Wd* is the weight of dry sponges. The experiment was performed in triplicate.

### 4.4. Structural Analysis

- *Circular Dichroism Spectroscopy*

The secondary structure of the perch collagen extracts was evaluated by circular dichroism spectroscopy (CD). Spectra acquisitions were performed on a Jasco J-1500 Spectrophotometer model, Tokyo, Japan (J-1500 Circular Dichroism Spectrophotometer) using a quartz cell with l = 10 mm.

For each reading, 500 µL of fish extracts aqueous solution of 0.05% concentration was used. The CD spectra were obtained by scanning in triplicate, using the spectral range 185–250 nm, with a scanning speed of 100 nm/min.

- *Fourier transform infrared spectroscopy (FTIR)*

Perch collagen extracts were subjected to FTIR spectral analysis using a Vertex 70 Bruker FTIR spectrometer (Bruker, Billerica, MA, USA). The following settings were used to record each FTIR spectrum in the ATR mode: resolution of 4 cm^−1^, spectral range of 4000–600 cm^−1^ and 30 acquisitions for every sample.

### 4.5. Collagen Scaffolds Microbiological Analysis

The samples were tested on ATCC strains from the collection of the ICPI Biotechnology Laboratory, namely, on *E. coli* (ATCC 10536, Mediclim, Bucharest, Romania) and *S. aureus* (ATCC 6538, Mediclim, Bucharest, Romania). The working method was diffusion method. A tube containing Mueller Hinton Broth (MHB, Novachim, Bucharest, Romania) inoculated with *E. Coli* and *S. aureus* was incubated for 18 h at 37 °C. Decimal dilutions up to 10^−5^ CFU/mL were made from this tube. An amount of 200 μL of the inoculum was seeded onto the plates. Afterward, the samples thus prepared were added and kept in the incubator for 24 h at 37 °C. Antibacterial activity was determined by measuring the diameter of the zone of inhibition around every sample. For microbial contamination, Casein Soya Bean Digest Agar (Novachim, Bucharest, Romania) was employed for total aerobic microbial count (TAMC) and Sabouraud Dextrose Agar (Novachim, Bucharest, Romania) for total fungal count.

### 4.6. Assessment of Biocompatibility of Collagen Scaffolds

In order to have a more complete characterization of the collagen scaffolds’ impact on human skin cells, the cytotoxicity of extracts obtained by incubating the samples in culture medium was tested, followed by assessment of capacity to support colonization with human dermal fibroblasts. As control, a collagen scaffold (COLL) without tartaric, tartaric plus citric and ascorbic acids addition was used.

The collagen scaffolds were incubated in complete culture medium (low glucose Dulbecco’s Modified Eagle Medium from Sigma Aldrich, St. Louis, MO, USA), supplemented with 10% (*v*/*v*) fetal bovine serum from Gibco BRL, Gaithersburg, MD, USA and 100 IU/mL penicillin, 100 µg/mL streptomycin and 50 µg/mL neomycin, all from Sigma Aldrich, St. Louis, MO, USA, at a 2 mg/mL ratio for 6 h/37 °C under stirring. Subsequently, the samples were clarified by centrifugation (300 g/5 min) and sterilized by filtration (0.2 μm pore size). Next, the effect of this extract on the viability of human adult fibroblasts was assessed in addition to the impact on cell function (migration capacity). The chemotactic properties of the tested freeze-dried wafers were assessed by xCELLigence RTCA system (Acea Biosciences, Inc). The human adult fibroblasts were obtained as previously described [50] and cultured in complete culture medium, at 37 °C and 5% CO_2_.

For the viability assessment, cells were seeded in complete medium on 96-well plates either in the presence of extracts (1 × 10^4^ cells/cm^2^) or on pre-cut collagen scaffolds (5 × 10^5^ cells/well). For this, sheets of scaffolds were cropped with a 4 mm in diameter punch, sterilized in 70% ethanol overnight, followed by washing in sterile water, and maintained in DMEM without serum for at least 24 h. The viability was evaluated in triplicates by XTT assay (Thermo Fisher Scientific, Waltham, MA, USA) 24, 48 and 72 h after the addition of the extracts or 5 days after the seeding of scaffolds.

The effect of collagen scaffold extracts on the fibroblast’s migration capacity was evaluated by scratch test. Thus, cells were seeded in complete medium on 96-well plates (1 × 10^4^ cells/cm^2^). Twenty-four hours before the test, cells were serum starved. A lesion was performed on the monolayer using a 200 μL pipette tip and the cells were incubated in the presence of extracts using 8 biological replicates. Pictures were taken immediately after adding the extracts (0 h) and 16 h later. The cell migration was evaluated by quantifying the area covered by the cells using the ImageJ 1.42q software (NIH, Bethesda, MD, USA). The results were presented as percentage of coverage of the scratched area.

The capacity of the tested collagen scaffolds to support colonization with dermal fibroblasts was further assessed. Thus, cells were seeded onto the cropped scaffolds as described above, and, 5 days later, the samples were fixed in 4% PFA and processed for paraffin embedding. Slices that were 5 µm thick were obtained by using a Leica microtome and subsequently subjected to hematoxylin–eosin staining. Briefly, the slices were incubated for 1 min with hematoxylin and 30 s with eosin Y, mounted in Shandon Consul-Mount (Thermo Fisher Scientific, Waltham, MA, USA) and visualized using a Zeiss Observer D1 microscope (Zeiss, Oberkochen, Germany).

The contraction of the scaffold was assessed by taking pictures of the cropped samples before seeding with cells and after 5 days in culture. The area of each sample was quantified by using the ImageJ software (NIH, Bethesda, MD, USA). Each picture was individually calibrated. The results were presented as percentage of the initial size at T0.

## Figures and Tables

**Table 1:** Proximate analysis of collagen gels extracted from perch skin.

Sample	Dry Substance Content, %	Ash Content, %	Total Nitrogen, %	Protein Content, %	pH, pH Units
GEL_COLL_T_rt_ *	3.41	undetectable	0.40	2.28	2.5
GEL_COLL_T_f_ **	1.85	undetectable	0.20	1.12	2.5
GEL_COLL_TC_rt_	3.44	undetectable	0.40	2.25	2.5
GEL_COLL_TC_f_	2.17	undetectable	0.21	1.18	2.5
GEL_COLL_A_rt_	2.14	undetectable	0.21	1.18	2.5
GEL_COLL_A_f_	2.58	undetectable	0.28	1.57	2.5

* rt = room temperature (samples kept at room temperature, at 15 °C); ** f = fridge (samples kept at refrigerator), where GEL_COLL_T represents samples treated with tartaric, GEL_COLL_TC represents samples treated with tartaric and citric acid, GEL_COLL_A represents samples treated with ascorbic acid.

**Table 2:** Determination of microbial contamination of collagen sponges.

Sample	Total Number of Aerobic Microorganisms (TAMC), CFU/g *	Total Number of Fungi and Filamentous Fungi (TYMC), CFU/g **	*E. coli* Absent	*S. aureus* Absent	*P. aeruginosa* Absent
COLL_T	5 CFU/g	3 CFU/g	absent	absent	absent
COLL_TC	6 CFU/g	2 CFU/g	absent	absent	absent
COLL_A	3 CFU/g	2 CFU/g	absent	absent	absent

* Admissibility conditions: Up to 100 CFU/g for topical products and up to 1000 CFU/g for pharmaceutical products; ** Admissibility conditions: Up to 100 CFU/g for topical products and for pharmaceutical products.

## Data Availability

The original contributions presented in the study are included in the article, further inquiries can be directed to the corresponding authors.
